# *Onchocerca volvulus* Mimicking Metastatic Breast Carcinoma

**DOI:** 10.4269/ajtmh.19-0740

**Published:** 2020-03

**Authors:** Victor E. Nava, Adetoun A. Ejilemele

**Affiliations:** 1 Department of Pathology, Veterans Affairs Medical Center, Pathology and Laboratory Medicine Service and George Washington University, Washington, District of Columbia;; 2 CMC Laboratory Services, University of Texas Medical Branch, Huntsville, Texas

**Figure 1.**
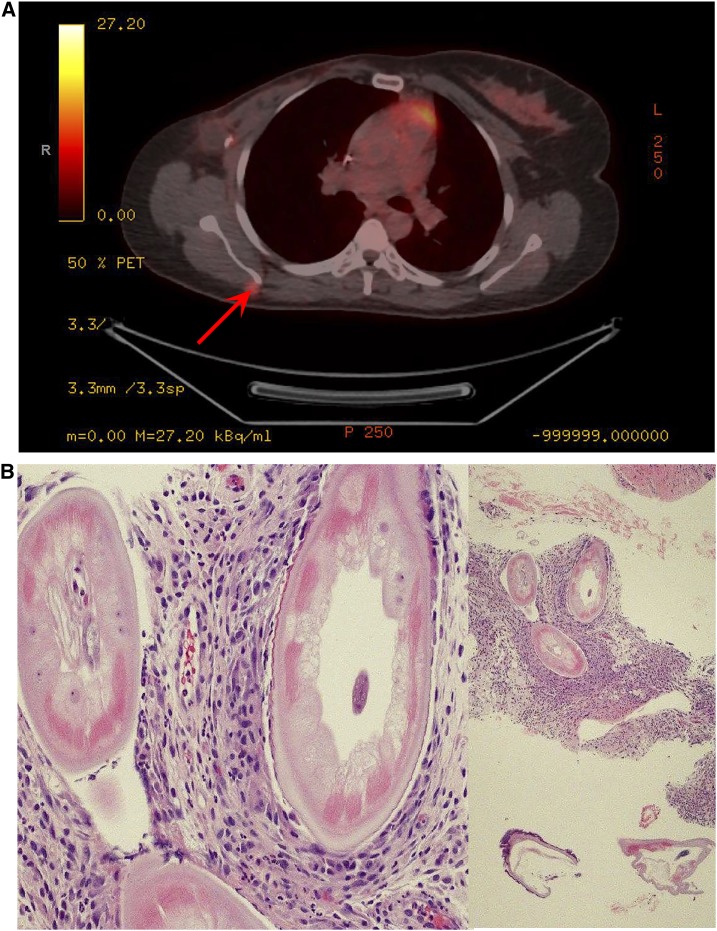
(**A**) FDG-avid lesion in the right periscapular region corresponding to a biopsied hypermetabolic soft tissue nodule. (**B**) Hematoxylin & Eosin-stained sections of the nodule showing *Onchocerca volvulus* (200× left and 40× right). This figure appears in color at www.ajtmh.org.

A 42-year-old Cameroonian woman with history of invasive ductal mammary carcinoma status post right mastectomy presented with a 1-cm right peri-scapular lesion. Position emission tomography scan showed a corresponding subcutaneous mildly fluorodeoxyglucose (FDG)-avid (standardized uptake value = 3.8) lesion, suspicious for metastasis (Figure 1A). Histologic examination of a skin biopsy revealed helminths consistent with *Onchocerca volvulus* (Figure 1B), and ivermectin was prescribed after infectology evaluation.

Onchocerciasis, a neglected tropical disease endemic in sub-Saharan Africa, Latin America, and the Middle East, is caused by penetration of the skin by third-stage filarial larvae when an infected blackfly (*Simulium* species) takes a blood meal. The larvae mature to adulthood in the subcutis and reside in nodules for up to 15 years. Female worms produce microfilariae that spread through lymphatics causing systemic manifestations, of which the most serious is blindness (“river blindness”). Repeated assault by infected blackflies is believed to facilitate infection in humans living near fast-flowing rivers. Transmission has not been reported in the United States, where the disease is exceedingly rare.

Onchocercomata rarely present in the breast.^1,2^ Interestingly, this is the first report in a breast cancer patient available in PubMed illustrating the importance of epidemiology.
